# Contributing factors to severe complications after liver resection: an aggregate root cause analysis in 105 consecutive patients

**DOI:** 10.1186/s13037-020-00261-7

**Published:** 2020-09-29

**Authors:** Kholoud Houssaini, Oumayma Lahnaoui, Amine Souadka, Mohammed Anass Majbar, Abdelilah Ghannam, Brahim El Ahmadi, Zakaria Belkhadir, Laila Amrani, Raouf Mohsine, Amine Benkabbou

**Affiliations:** 1 Surgical Oncology Department, National Institute of Oncology, Mohammed V University in Rabat, Rabat, Morocco; 2 Intensive Care Department, National Institute of Oncology, Mohammed V University in Rabat, Rabat, Morocco

**Keywords:** Aggregate root cause analysis, Liver resection, Postoperative complications, Patient safety

## Abstract

**Background:**

The aggregate root cause analysis (AggRCA) was designed to improve the understanding of system vulnerabilities contributing to patient harm, including surgical complications. It remains poorly used due to methodological complexity and resource limitations. This study aimed to identify the main patterns contributing to severe complications after liver resection using an AggRCA.

**Methods:**

This was a retrospective qualitative study aimed to identify the main patterns contributing to severe complications, defined as strictly higher than grade IIIa according to the Clavien-Dindo classification within the first 90 days after liver resection. All consecutive severe complications that occurred between January 1st, 2018 and December 31st, 2019 were identified from an electronic database and included in an AggRCA. This included a structured morbidity and mortality review (MMR) reporting tool based on 50 contributory factors adapted from 6 ALARM categories: “Patient”, “Tasks”, “Individual staff”, “Team”, “Work environment”, and “Management and Institutional context”. Data resulting from individual-participant root cause analysis (RCA) of single-cases were validated collectively then aggregated. The main patterns were suggested from the contributory factors reported in more than half of the cases.

**Results:**

In 105 consecutive liver resection cases, 15 patients (14.3%) developed severe postoperative complications, including 5 (4.8%) who died. AggRCA resulted in the identification of 36 contributory factors. Eight contributory factors were reported in more than half of the cases and were compiled in three entangled patterns: (1) Disrupted perioperative process, (2) Unplanned intraoperative change, (3) Ineffective communication.

**Conclusion:**

A pragmatic aggregated RCA process improved our understanding of system vulnerabilities based on the analysis of a limited number of events and a reasonable resource intensity. The identification of patterns contributing to severe complications lay the rationale of future contextualized safety interventions beyond the scope of liver resections.

## Background

For the past two decades, the global diffusion of modern liver resection techniques and evidence-based perioperative care practices has contrasted with the persistence of significant safety outcome disparities, including the incidence of severe postoperative complications and death rates [1]. To refine our understanding of the mechanisms contributing to patient harm, the adoption of strategies beyond the quantitative factors that revolve around the patient, the surgeon, and the surgical procedure is advocated [2].

Initially derived from high-hazard engineering industries, the root cause analysis (RCA) is a quality improvement strategy that focuses on system vulnerabilities that contribute to the likelihood of errors, rather than individual errors themselves. For that, it has become a standard tool to review single-case reports of adverse events across all healthcare specialties [3]. Recently, the aggregation of data from single-case RCAs (Aggregate RCA) was proposed to enhance insight into system functioning [4] and to refine the prioritization of interventions that would prevent the occurrence of similar events [5].

Despite its promising design to improve overall patient safety, the aggregate RCA remains rarely used to investigate surgical outcomes. In the specific field of hepatobiliary and pancreatic surgery, RCAs of postoperative deaths have revealed consistent patterns of contributory factors, including complication management delays, intraoperative technical incidents, and gaps in compliance with guidelines [6, 7]. These findings that were based on retrospective data aggregation from multiple centers should be challenged by the use of frameworks that extend and deepen the analysis of adverse outcomes to a wide scope of possible influences, including human factors [8, 9]. The ALARM framework, originally inspired by Reason’s model of organizational accidents [10, 11], was adapted to medicine to enable researchers to formalize such an approach [2].

The aim of this study was to use an aggregate RCA based on the ALARM framework to identify the main patterns of contributory factors associated with severe complications after liver resection in the setting of a North African anticancer center.

## Methods

The reporting of the research was made according to the consolidated criteria for reporting qualitative research (COREQ) checklist [12] and the manuscript was written according to the SQUIRE 2.0 guidelines [13].

The study was conducted in the surgical department at the National Institute of Oncology (NIO), which is an academic anticancer center in Rabat (Morocco, North Africa) with approximately 300 major abdominal surgeries, including 50 liver resections per year. The RCA was performed using a standardized reporting tool that was developed from the ALARM framework [2, 14] by the local multidisciplinary team to ensure that critical contributory factors are considered during morbidity and mortality reviews (MMRs). The tool has been used locally since July 2019 for weekly MMRs that are dedicated to investigating severe postoperative complications and near-misses. Therefore, participants in the study were familiar with the tool. The MMR reporting tool consists of 50 questions (Q) selected from a large set of examples from the commented ALARM framework proposed by the French High Authority for Health (Appendix 1). Each question investigates one of the contributory factors related to the six following ALARM categories: “Patient”, “Tasks”, “Individual staff”, “Team”, “Work environment”, and “Management and Institutional context”. This latter was obtained from the merge of two categories “Organizational and management factors” and “Institutional context factors”, as it was suggested by Vincent et al. ([2, 14]). Answers incriminating a contributing factor (“yes” or “no” depending on the context) are referred to as “triggered answer” or “triggered contributory factor”, indifferently. A “refuted” option or a “non-applicable (NA)” option (when information is judged lacking) is offered otherwise. Justifications and comments regarding triggered contributory factors, recovery factors, and corrective measures are included in the final report of the MMR. The set of 50 questions of the MMR reporting tool and their ALARM categorization are presented in Appendix 2.

In the current study, an aggregate RCA (AggRCA) based on the ALARM framework [2] was used as a method to identify the main patterns of contributory factors associated with severe complications after liver resection. A pattern was defined as a regular sequence of factors contributing to the predefined outcome (vs. single root cause [5]).

In order to limit data overwriting, we chose to analyze aggregated data from independent RCAs of single cases, rather than making a root cause analysis directly from a summary of the cohort.

All the cases of severe complications after elective liver resection that were consecutively performed at an academic surgical department between January 1st, 2018 and December 31st, 2019 were included. Severe complications were defined as complications strictly higher than grade IIIa according to the Clavien-Dindo classification within the first 90 postoperative days (PODs) [15, 16].

In order to overcome selection and availability biases associated with voluntary reporting of adverse events [17], cases were identified from an electronic database including all liver resections performed at the department.

The research team included the surgeon in charge of the liver surgery program at the NIO (BA), a surgical resident (LO), and a research fellow that acted as a third party (HK). Six clinicians (4 surgeons, 2 intensivists) and 2 nurses, were purposively selected among surgical and intensive care staff given their involvement in the management of liver resections and their experience with mortality and morbidity reviews (MMRs). Characteristics and roles of participants and research team members are detailed in Table 1.

**Table 1:** Characteristics and roles of the research team and study participants

Initials, Credentials	Age, Gender	Specialty (subspeciality), Current position at the NIO	Experience in the specialty; Experience at the NIO**	Roles in the Aggregate Root Cause Analysis (RCA) process				
				Step 1 Event storyline	Step 2 Single-case RCAs	Step 3 Step 2 consolidation	Step 4 Focus group Step 3 validation	Step 5 Aggregate RCA
HK*	26 years, Female	MD student, Research fellow	NA; 24 months	Production	NA	Participation	Co-facilitation	Participation
LO*, MD	27 years, Female	Surgery, Resident	2 years; 18 months	Production	Participation	Participation	Participation	Participation
BA*, MD	41 years, Male	Surgery (hepatobiliary), Attending physician, MMR coordinator	10 years; 25 months	Validation	Participation	Participation	Facilitation	Participation
**GA, MD**	36 years, Male	Anesthesiology & Intensive care, Attending physician, MMR coordinator	7 years; 59 months	Validation	Participation	NA	Participation	NA
**EB, MD**	37 years, Male	Anesthesiology & Intensive care, Attending physician	7 years; 31 months	NA	Participation	NA	Participation	NA
**MA, MD**	40 years, Male	Surgery (colorectal), Attending physician	10 years; 20 months	NA	Participation	NA	Participation	NA
**SA, MD**	39 years, Male	Surgery (colorectal, peritoneal surface), Attending physician, Head of the OR	8 years, 64 months	NA	Participation	NA	Participation	NA
**AM**	38 years, Male	Nurse, Head nurse	13 years, 157 months	NA	NA	NA	Participation	NA
**AS**	29 years, Female	Nurse, Patient care coordinator	6 years, 27 months	NA	NA	NA	Participation	NA

^*^ Research team

^**^At the end of the study

*MD* medical doctor, *NA* not applicable, *NIO* National Institute of Oncology, *OR* operative room

The AggRCA was conducted through a five-step process over the period from December 2019 to March 2020. The MMR reporting tool was used for data collection and aggregation.

### Step 1: event storyline

For each case, a storyline depicting the timeframe of the perioperative care was created. Data were collected from the electronic database and completed from the patients’ respective hard copy files: case history, physical examination, results and/or copies of documented preoperative medical imaging, pre-anesthetic consultation reports, treatment plan decisions, procedure reports, monitoring, and complication diagnosis and management. Interviews with staff members were conducted in case of missing information to obtain the most comprehensive case reports.

### Step 2. Single-case RCAs

Anonymized storylines were emailed to the six participating clinicians individually for review. Each participant was asked to fill out the MMR reporting tool for every single case independently to determine potential contributory factors, recovery factors, and corrective measures. A deadline was set for two weeks after receipt of the storylines.

### Step 3. Consolidation of single-case RCAs (workshop)

For each question, the research team consolidated the respondents’ answers into a single option: “Triggered”,” Refuted” and “NA”. Consolidation was based on the congruence of responses between at least three (half) of the respondents, unless a justification that was presented brought unique insights: individual staff perception (stress, fatigue, moral support) and/or specific perspective of the context of events (direct involvement in the management in the ICU or the OR). Conflicting justifications were identified and deferred to the next step for resolution. Recovery factors and corrective measures were then pooled together.

### Step 4. Validation of single-case RCAs (focus group)

All the participants were gathered one month after the individual analysis was completed. The consolidation process and results were presented. Conflicting justifications were discussed and settled on a case by case basis until a consensus was reached for all triggered contributory factors, recovery factors, and corrective measures.

### Step 5. Aggregate RCA

Combinations of triggered contributory factors across the single cases were visualized according to the ALARM categories. Validated data from single cases were aggregated to obtain a distribution (percentage) of triggered contributory factors and their respective categories among the whole cohort.

A network of relationships (contribution to harm and/or failure to prevent harm) was established between the contributory factors that were triggered in more than half of the cases. The main patterns were suggested from the network analysis and refined from the integration of less frequent contributory factors, as well as insights from recovery factors and corrective measures.

#### Statistical analysis

Aggregated data from single-case answers were summarized into descriptive statistics tables, including median, percentages, standard deviation, and quartiles when appropriate. Analyses were performed using Google sheet.

## Results

### Description of the study population

15 patients from the study population in 105 consecutive cases of elective liver resections developed severe complications. These patients therefore met the inclusion criteria to be analyzed according to the five-step process. The severe complication rate within 90 days of index surgery was 14.3%, including a mortality rate of 4.8%.

All the cases underwent a liver resection for a malignant lesion: colorectal liver metastasis (*n* = 8, 53.3%), gallbladder cholangiocarcinoma, (*n* = 2, 13.3%), perihilar cholangiocarcinoma (n = 2, 13.3%), hepatocellular carcinoma (n = 2, 13.3%); neuroendocrine tumor liver metastasis (*n* = 1, 6.7%). Major liver resection was performed in six cases. Major visceral (colectomy, proctectomy, gastrectomy, main bile duct, and/or biliary confluence), diaphragmatic, and/or vascular resections were associated with liver resection in 7 cases (46.7%), 2 cases (13.3%), and 1 (6.7%) case, respectively. Causes of death (*n* = 5) were multifactorial and involved pleuro-pulmonary sepsis, abdominal sepsis from the biliary or digestive origin (n = 2, 40%), and liver failure (n = 1, 20%). A summary of demographics and clinical data of the 15 cases is shown in Table 2. There was no missing data in this study.

**Table 2:** Demographics and clinical data of the 15 single cases

Case	Sex, Age	BMI	Indication	Liver resection Type	Associated procedure	Operative time, Minutes	Estimated blood loss, mL	Total Pringle time, Minutes	Type of severe complication	Relaparotomy, (Number)	Clavien-Dindo Grade	Time from Liver resection to death, Days
**1**	M, 50	30.00	GBCC	Bisegmentectomy Sg4b-Sg5	Main bile duct resection / Hepaticojejunostomy	240	200	40	Severe sepsis secondary to subphrenic abscess	No	IVa	NA
**2**	F, 53	25.53	GBCC	Bisegmentectomy Sg4b-Sg5	Distal Gastrectomy / Gastrojejunostomy	300	50	20	Biliary peritonitis secondary to cut surface leak	Yes	IIIb	NA
**3**	F, 79	29.61	CRLM	Bisegmentectomy Sg6-Sg7	No	150	150	48	Death caused by arrhythmia associated to septic pleural effusion	No	V	37
**4**	M, 73	23.30	CRLM	Wedge resections (4) in Sg6-Sg8, Sg4a, Sg3 and Sg2	No	330	300	79	Delayed awakening following anesthesia and delayed weaning POD4 associated to hemodynamic instability	No	IVa	NA
**5**	F, 68	26.63	HCC	Right hepatectomy with resection of Sg1	Diaphragm resection	330	400	0	Acute respiratory failure secondary to pulmonary embolism	No	IVa	NA
**6**	M, 61	21.97	CRLM	Wedge resection in Sg5	Right colectomy / Ileocolic anastomosis	180	50	15	Hemorrhagic shock secondary to ruptured false aneurysm / Biliary Peritonitis secondary to cut surface leak	Yes (2)	IVa	NA
**7**	M, 70	26.64	NETLM	Left hepatectomy with resection of Sg1	No	300	250	45	Hemorrhagic shock secondary to cut surface bleeding	Yes	IVa	NA
**8**	F, 51	23.87	CRLM	Wedge resections (2) in Sg4 and Sg5	Proctectomy / Colorectal Anastomosis	310	50	0	Death caused by septic shock secondary to a peritonitis from anastomosis leak	Yes	V	4
**9**	M, 31	19.09	CRLM	Left hepatectomy with wedge resections (3) in Sg1, Sg5-Sg6 and Sg6-Sg7	No	300	200	13	Biliary peritonitis secondary to a cystic duct leak	Yes	IIIb	NA
**10**	M, 60	29.41	CRLM	Wedege resection in Sg8	No	240	100	30	Acute respiratory failure secondary to pneumonia	No	IVa	NA
**11**	F, 58	26.45	PHCC	Left hepatectomy with resection of Sg1	Biliary confluence resection / Hepaticojejunostomy	420	700	12	Death caused by sepsis secondary to pneumonia, associated to liver failure	No	V	13
**12**	F, 57	31.36	CRLM	Segmentectomy Sg7	Proctectomy / Colorectal anastomosis	240	300	30	Peritonitis secondary to anastomosis leak	Yes (2)	IIIb	NA
**13**	F, 54	22.83	CRLM	Left hepatectomy with wedge resection (2) in Sg7 and Sg4b	Diaphragm and pericardium resection	300	700	26	Arrhythmia secondary to a pneumopericardium, associated to kidney failure	No	IVa	NA
**14**	M, 63	25.43	HCC	Segmentectomy Sg5	No	240	150	29	Death caused by sepsis secondary to pneumonia	No	V	5
**15**	M, 53	25.00	PHCC	Left hepatectomy with resection of Sg1	Biliary confluence resection / Hepaticojejunostomy Portal resection / End to end anastomosis	360	500	32	Death caused by septic shock secondary to cholangitis	No	V	17
*BMI* body mass index, *CRLM* colorectal liver metastasis, *GBCC* gallbladder cholangiocarcinoma, *HCC* hepatocellular carcinoma, *NETLM* neuroendocrine tumor liver metastasis, *PHCC* perihilar cholangiocarcinoma, *Sg* segment, *RBC* red blood cells, *ICU* intensive care unit												

### The aggregate root cause analysis process

**Fig. 1:**
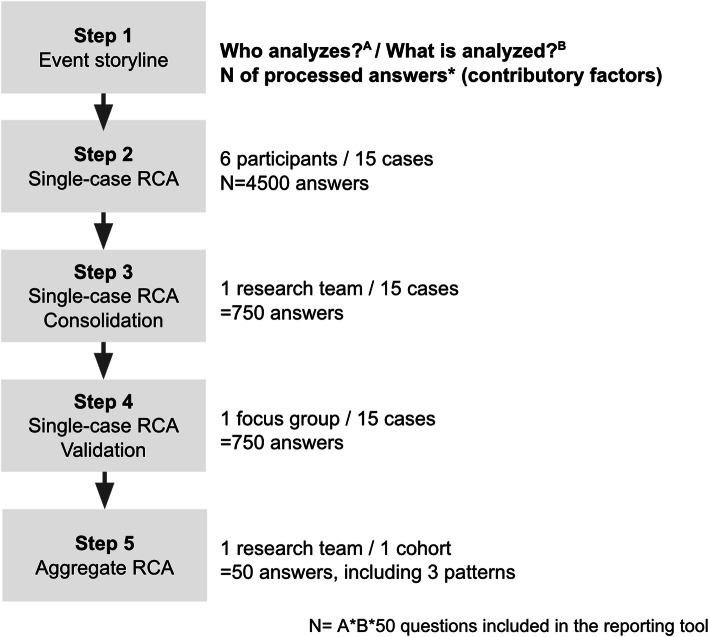
Processing of the answers to the MMR reporting tool across the aggregate RCA steps

The processing of the answers to the MMR reporting tool in the five steps of the AggRCA process is shown in Fig. 1.

#### Step 1 and 2. Single-case RCAs

All six participating clinicians sent back fully filled out RMM reporting tools regarding all the 15 cases.

#### Step 3. Consolidation of single-case RCAs

Consolidation (from 4500 to 750 answers) was based on the congruence of responses between at least three respondents in 662 (88.2%) cases and a unique insight in 76 (10.2%) cases. In 12 (1.6%) cases, consolidation was not resolved at this step because of conflicting justifications.

#### Step 4. Validation of single-case RCAs

All conflicting justifications were resolved. The consensus was reached for each triggered contributory factor, recovery factor, and corrective measure. For each case, a median of 10 contributory factors was triggered (extremes 4–20).

#### Step 5. Aggregate RCA

Combinations of contributory factors incriminated in the 15 single cases, according to ALARM categories are shown in Table 3.

**Table 3:**
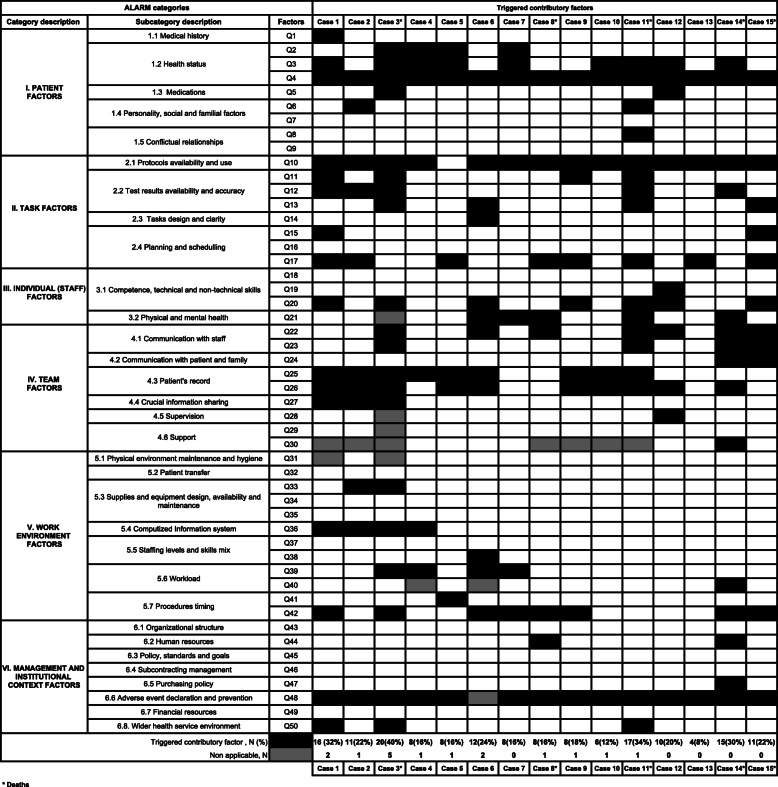
Combinations of triggered contributory factors across the 15 single cases

Overall, 36 contributory factors (72%) within all six ALARM categories were triggered at least once across the 15 cases. The main triggered categories were: “Task factors” (31.6%), “Team factors” (25%), “Patient factors” (24.4%), and “Individual staff factors” (21.7%). Eight contributory factors were triggered in more than half of the cases: “Patient health conditions”(Q3, 53.3%), “Complexity of the case” (Q4, 100%), “Protocols availability and use” (Q10, 93.3%), “Intraoperative strategy change” (Q17, 60%), “Accessibility and completeness of the medical records” (Q25, 60%), “Risk highlights within the medical records” (Q26, 66.6%), “Complication management delays” (Q42, 53.3%) and institutional “Adverse event declaration and prevention” (Q48, 93.3%). Details of triggered combinations of contributory factors across the 15 single cases and their distribution among the whole cohort are presented in Tables 3 and 4, respectively.

**Table 4:** Distribution of triggered contributory factors among the cohort

ALARM categories			Distribution of triggered contributory factors	
**Category description**	**Subcategory description**	**Factors**	**Per question N (%)**	**Per category %**
**I. PATIENT** **FACTORS**	1.1 Medical history	Q1	1 (6.6%)	**24.4%** (33/135)
	**1.2 Health status**	Q2	4 (26.6%)	
		**Q3**	**8 (53.3%)**^a^	
		**Q4**	**15 (100%)**^a^	
	1.3 Medications	Q5	2 (13.3%)	
	1.4 Personality, social and familial factors	Q6	2 (13.3%)	
		Q7	0 (0%)	
	1.5 Conflictual relationships	Q8	1 (6.6%)	
		Q9	0 (0%)	
**II. TASK** **FACTORS**	**2.1 Protocols availability and use**	**Q10**	**14 (93.3%)**^a^	**31.6%** (38/120)
	2.2 Test results availability and accuracy	Q11	4 (26.6%)	
		Q12	5 (33.3%)	
		Q13	4 (26.6%)	
	2.3 Tasks design and clarity	Q14	1 (6.6%)	
	**2.4 Planning and scheduling**	Q15	2 (13.3%)	
		Q16	0 (0%)	
		**Q17**	**8 (53.3%)**^a^	
**III. INDIVIDUAL (STAFF) FACTORS**	3.1 Competence, technical and non-technical skills	Q18	0 (0%)	**21.7%** (13/60)
		Q19	1 (6.6%)	
		Q20	7 (46.6%)	
	3.2 Physical and mental health	Q21	5 (33.3%)	
**IV. TEAM FACTORS**	4.1 Communication with staff	Q22	7 (46.6%)	**25.2%** (34/135)
		Q23	3 (20%)	
	4.2 Communication with patient and family	Q24	2 (13.3%)	
	4.3 Patient record	Q25	**9 (60%)**^a^	
		Q26	**10 (66.6%)**^a^	
	4.4 Crucial information sharing	Q27	1 (6.6%)	
	4.5 Supervision	Q28	1 (6.6%)	
	4.6 Support	Q29	0 (0%)	
		Q30	1 (6.6%)	
**V. WORK ENVIRONMENT FACTORS**	5.1 Physical environment maintenance and hygiene	Q31	0 (0%)	**11.7%** (21/180)
	5.2 Patient transfer	Q32	0 (0%)	
	5.3 Supplies and equipment design, availability and maintenance	Q33	2 (13.3%)	
		Q34	0 (0%)	
		Q35	0 (0%)	
	5.4 Computized Information system	Q36	4 (26.6%)	
	5.5 Staffing levels and skills mix	Q37	0 (0%)	
		Q38	1 (6.6%)	
	5.6 Workload	Q39	4 (26.6%)	
		Q40	1 (6.6%)	
	**5.7 Procedures timing**	Q41	1 (6.6%)	
		**Q42**	**8 (53.3%)**^a^	
**VI. MANAGEMENT AND INSTITUTIONAL CONTEXT FACTORS**	6.1 Organizational structure	Q43	0 (0%)	**16.6%** 20/120
	6.2 Human resources	Q44	2 (13.3%)	
	6.3 Policy, standards and goals	Q45	0 (0%)	
	6.4 Subcontracting management	Q46	0 (0%)	
	6.5 Purchasing policy	Q47	1 (6.6%)	
	**6.6 Adverse event declaration and prevention**	**Q48**	**14 (93.3%)**^a^	
	6.7 Financial resources	Q49	0 (0%)	
	6.8. Wider health service environment	Q50	3 (20%)	

^a^Factors incriminated in more than half of the cases

17 recovery factors within five ALARM categories were reported across the 15 cases. Three recovery factors were noted in more than half of the cases: “Family support” (66.6%), “Shared decision by the surgical team” (60%), and “Concertation between surgery and ICU teams” (53.3%).

24 corrective measures within all six ALARM categories were suggested. Only two of them (8.3%) were specifically related to liver resection techniques. Ten (42%) measures including all those related to the “Patient factors” consisted of recommendations to implement or reinforce protocols. Details of the characteristics of recovery factors and corrective measures are presented in Table 5.

**Table 5:** Characteristics of recovery factors and corrective measures among the cohort

ALARM category	Recovery factors		Corrective measures	
	N	Description (n associated cases)	N	Description
**I. PATIENT** **FACTORS**	**2**	- Family support (10 cases) - Proactive adaptation of intraoperative support to case complexity (6 cases)	**5**	- To restrict the indications of combined colorectal surgery** - To implement a protocol for patient psychological assessment** - To implement a protocol for patient oncogeriatric assessment** - To implement a protocol for patient nutritional assessment** - To implement a management protocol for obese patients
**II. TASK FACTORS**	**6**	- Proactive readmission to the ICU (5 cases) - Proactive indication of imaging (4 cases) - Proactive revision surgery to control complication (4 cases) - Management of the complication by attendings (2 cases) - Proactive indication of percutaneous drainage (1 case) - Complication management handover (1 case)	**5**	- To implement a protocol for intraoperative changes in strategy - To mention treatment strategy changes in surgical report - To implement protocols for operating instructions of medical devices **- To implement management protocols for liver resection complications:** **- percutaneous drainage** **- pleural effusion** **- anastomosis leaks** - To implement postoperative management protocols: - indications of imaging** - emergency revision surgery (management and supervision) - criteria for hospital discharge** - criteria for ICU discharge**
**III. INDIVIDUAL (STAFF)** **FACTORS**	**4**	- Proactive call for intraoperative surgical support (6 cases) - Proactive hemorrhage management by a resident (2 cases) - Proactive hemorrhage management by a nurse (1 case) - Complication management handover (1 case)	**3**	- To check cystic ligature after hepatic pedicle clamping** - To check the loss of selectivity of the clamping during liver section** - To discuss a validation for change in intraoperative strategy
**IV. TEAM** **FACTORS**	**4**	- Shared decision by the surgical team (9 cases) - Internal multidisciplinary concertation: Surgery-ICU (8 cases) - External multidisciplinary concertation, e.g.: Thoracic surgery (3 cases) - Proactive revision surgery to control hemorrhage (1 case)	**3**	- To offer insight when validation for change in intraoperative strategy - To optimize internal communication (Surgery-ICU)** - To optimize external communication (Outside of NIO)**
**V. WORK ENVIRONMENT** **FACTORS**	**0**		**4**	- To adapt workload during holiday seasons** - To optimize nurses’ night on-call scheduling - To optimize records of medical and paramedical procedures** - To implement a system of patient risk management
**VI. ORGANIZATIONAL AND MANAGEMENT FACTORS**	**1**	- Immediate availability of blood* (1 case)	**3**	- To tackle the failure of bacteriology test circuit - To report MMR recommendations to the hospital administration - To tackle the issue of blood shortage
**Total**	**17**	**Total**	**23**	

* Near miss, **Ongoing improvement

in Bold: specific to liver resection

*BMI* body mass index, *ICU* Intensive Care Unit, *NIO* National Institute of Oncology

### Patterns contributing to severe complications

**Fig. 2:**
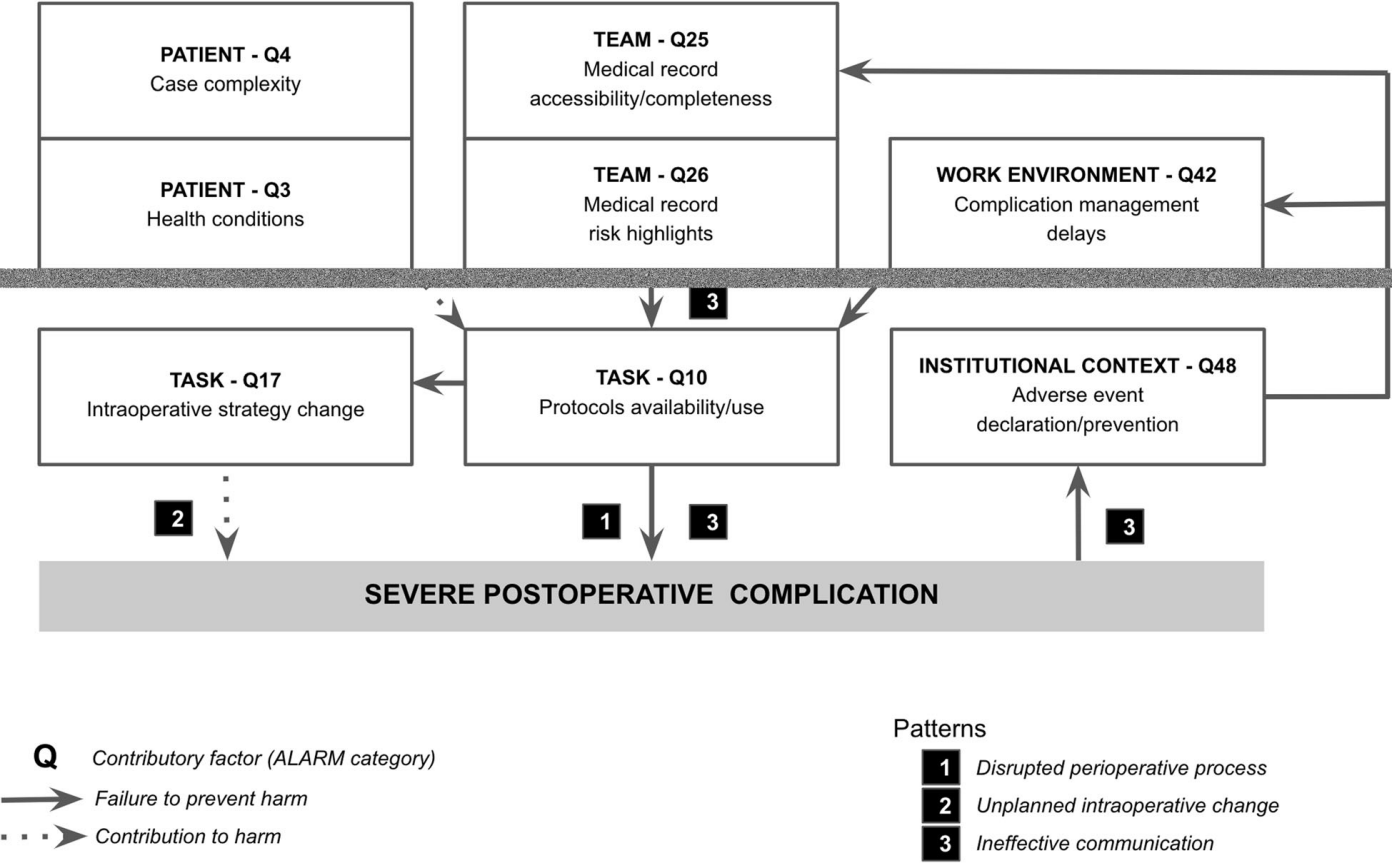
Main patterns contributing to severe postoperative complication after liver resection

Three entangled patterns were suggested from the network of relationships built from the eight most frequent factors contributing to the occurrence of severe complications after liver resection. These are shown in Fig. 2.

#### Pattern 1: disrupted perioperative process

Nonoptimal protocol availability and/or use was consistently reported (Q10, 93.3%), especially in the assessment of patients with preexisting health conditions (Q3, 53.3%), such as advanced age, obesity, altered nutritional status, and mental health issues. Delayed diagnosis and/or treatment of complications (Q42, 53.3%) was attributed to intra-team factors, such as clinical hesitations (imaging indication, revision surgery indication) and systemic factors such as regional blood shortage, lab test dependency upon a distant hospital, and senior radiology staff unavailability. Intra-team contributory factors may have been influenced by individual stress and/or fatigue (Q21 33.3%) and heavy clinical workloads (Q39, 26.6%) that were reported when many complex cases and complications were dealt with during the same period. This called for workforce management and surgical scheduling adaptation upon security standards.

#### Pattern 2: unplanned intraoperative change

An unplanned extension of the resection to liver parenchyma or another organ was performed as a technical adaptation in order to achieve tumor-free resection margins (Q17, 60%). This was associated with postoperative deaths in more than half of the cases. Although reflecting the complexity (Q4, 100%) of many cases (large and/or multiple infiltrative tumors with borderline resectability), the performance of unplanned procedures called for the implementation of a formal intraoperative decision-making process. In two cases (unplanned portal resection and unplanned major liver resection associated with multiple wedge resections), strategy change has favored intraoperative incidents, which prompted the recommendation of technical correctives measures (e.g: selectivity check of portal clamping during liver section, ligation check of the cystic duct after multiple pringle maneuvers).

#### Pattern 3: ineffective communication

This pattern was involved at four levels. First, previous similar adverse events were not communicated (Q 48, 93.3%) to other hospital staff or institutional departments such as quality improvement committees and hygiene committees. This precluded the possibility to address systemic issues involved in patterns 1 and 2. Second, written communication through medical records was often lacking (Q25, 60%) and not drawing enough attention to preventable risks (Q26, 66.6%). Third, intra-team miscommunication was reported in nearly half of the cases (Q22, 46.6%). However, crucial information was always shared between staff members (Q27). Finally, inter-team ineffective communication issues were less frequently reported (Q23, 20%), but it occurred in one case of postoperative death that may have been prevented if a timely transfer of the patient from another city was achieved.

## Discussion

In the current study, an aggregate RCA provided a systemic description of harm mechanisms after liver resection in the setting of a tertiary anticancer center in Morocco (North Africa). The analysis revealed 36 contributory factors covering the full range of the ALARM categories, 17 recovery factors that have potentially limited the evolution of complications towards greater severity, and 23 corrective measures. The analysis identified three major entangled patterns of contributory factors that were put into perspective with corresponding recovery factors and corrective measures: (1) “Disrupted perioperative process”, (2) “Unplanned intraoperative change”, and (3) “Ineffective communication”.

In this cohort, the 90-day postoperative mortality rate was < 5%, which is in line with the results of expert centers and western national registries [18, 19]. A high prevalence of sepsis among severe complications (versus liver failure) and a short interval between index surgery and death compared to series from expert centers were reported [20]. This should be investigated, while acknowledging latent factors that are common to developing countries, such as disruption to intensive care services and blood shortage [21].

In this study, we chose to identify patterns of contributory factors rather than a single or a small number of root causes. This allows us to better describe the entanglement of active failures and latent conditions [22], and consider the dynamics of their interactions [23].

 In the medical literature, most studies that tackle “Disruptions in perioperative processes” (Pattern 1) refer to deviations in the respect of guidelines and/or local protocols [6]. In a previous RCA study of 86 postoperative deaths after liver resection, guidelines and postoperative management protocols were not respected in 57 and 22% of the cases, respectively [20]. Although it is critical, the implementation of measures to ensure compliance with evidence-based practice may be challenged by systemic factors depending on the context (e.g: blood and drug shortages, limited access to imaging, and lab tests). These factors may lead to individual and team compensation mechanisms and may exacerbate the stress and burnout associated with the management of complications and the second victim syndrome [24, 25]. In the present study, individual staff proactivity and family support (regardless of socioeconomic status) were the most frequently reported recovery factors that may reflect compensation mechanisms to systemic failures [26].

Unplanned intraoperative change (Pattern 2) involves a cascade of events that favor the violation of intraoperative guidelines and the occurrence of technical errors. Tumor progression leading to a more extensive procedure than planned is a typical pattern of postoperative complication and death [20]. Stress, cognitive biases such as the sunk cost fallacy and the anchoring effect [17], and overconfidence in one’s intuition [27] may explain why it is challenging for surgeons to process significant updates in the balance between safety and potential oncological benefits. This underscores the importance of preoperative planning including up-to-date imaging, multidisciplinary assessment, and accurate evaluation of remnant liver volume when indicated. In the event of unforeseen intraoperative findings, a break in the operative course [20] and discussion with colleagues (surgeons, intensivists, oncologists) have been suggested to prevent futile and potentially lethal surgeries [20, 28–30].

Ineffective communication (Pattern 3) covers a spectrum of situations that extends from oral interpersonal communication to written traceability in the medical records. It may be maintained by a culture of blame and low empowerment to notify disagreements and institutional failures, [8, 31]. The communication of RCA results to key stakeholders and other staff [3] is a lever for tackling systemic factors and promoting a safety culture. It may scale up harm mitigation and support the sustainability of effective corrective measures [32].

In the current study, actionable system vulnerabilities were revealed by a collaborative methodology that allowed us to draw a maximum of relevant information from a limited number of events.

Effective multidisciplinary staff participation was favored by capitalizing on a pre-existing MMR process including a structured reporting tool. As it was already suggested, the MMR, which is a regulatory obligation in many countries, may represent an alternative to overcome the limited methods and intensity of resources (time, human and financial) to conduct a formal RCA [33, 34, 35]. The use of a common taxonomy (ALARM framework) for the contributory factors, the recovery factors, and the corrective measures supported a comprehensive approach to patterns identification and improvement strategies recommendations.

### Limitations

This study has some limitations. First, the inclusion of random cases of liver resection that were not followed by a complication may have overcome hindsight bias and reveal more latent contributory factors [36]. Second, the aggregation of a limited number of cases across a two-year period may have overlooked other contributory factors and potential evolutions of patterns. This invites us to keep an open mind on system changes including the collective learning curve and consider the need to update our interpretations. Third, emotional bias due to the involvement of participants in the management of the cases could not be totally excluded, despite the participation of a third party (HK). However, we believe on the contrary that the inclusion of experts in the concrete functioning of the studied system associated with a methodology based on formal justification added value to our approach.

Finally, an improved analysis may have been limited by inaccuracies related to the selection of questions of the MMR reporting tool and their formulation. Inclusion of a more relevant guidelines/protocols subdivision (cancer-related, patient-related, and procedure-related) and human behavior categorization (knowledge-based, rule-based, and skill-based) [37, 38] should be undertaken.

## Conclusion

In this study, a pragmatic aggregated RCA methodology resulted in the identification of patterns contributing to severe complications after liver resection, based on the study of a limited number of events and a reasonable resource intensity. It revealed system vulnerabilities and potential safety interventions that may be exploited beyond the scope of liver surgery.

Future studies from different settings and subspecialty backgrounds are needed to examine the applicability of current methodology for conducting, aggregating, and analyzing data from RCAs of postoperative complications.

## Supplementary information

**Additional file 1.** Commented ALARM framework as proposed by the French High Authority for Health.

**Additional file 2.** The 50 questions of the MMR reporting tool and their categorization according to the ALARM framework.

## Data Availability

Not applicable.

## Abbreviations

AggRCA: Aggregate root cause analysis
MMR: Morbidity and mortality review
NA: Non-applicable
NIO: National Institute of Oncology
POD: Post-operative day
RCA: Root cause analysis
