# Correction to: Contributing factors to severe complications after liver resection: an aggregate root cause analysis in 105 consecutive patients

**DOI:** 10.1186/s13037-020-00268-0

**Published:** 2020-11-12

**Authors:** Kholoud Houssaini, Oumayma Lahnaoui, Amine Souadka, Mohammed Anass Majbar, Abdelilah Ghannam, Brahim El Ahmadi, Zakaria Belkhadir, Laila Amrani, Raouf Mohsine, Amine Benkabbou

**Affiliations:** 1 Surgical Oncology Department, National Institute of Oncology, MohammedV University in Rabat, Rabat, Morocco; 2 Intensive Care Department, NationalInstitute of Oncology, Mohammed V University in Rabat, Rabat, Morocco

**Correction to: Patient Saf Surg 14, 36 (2020)**

**https://doi.org/10.1186/s13037-020-00261-7**

Following publication of the original article [1], the author would like to make the below two corrections:

- Change Table 3 (with shaded color).

**Table 3:**
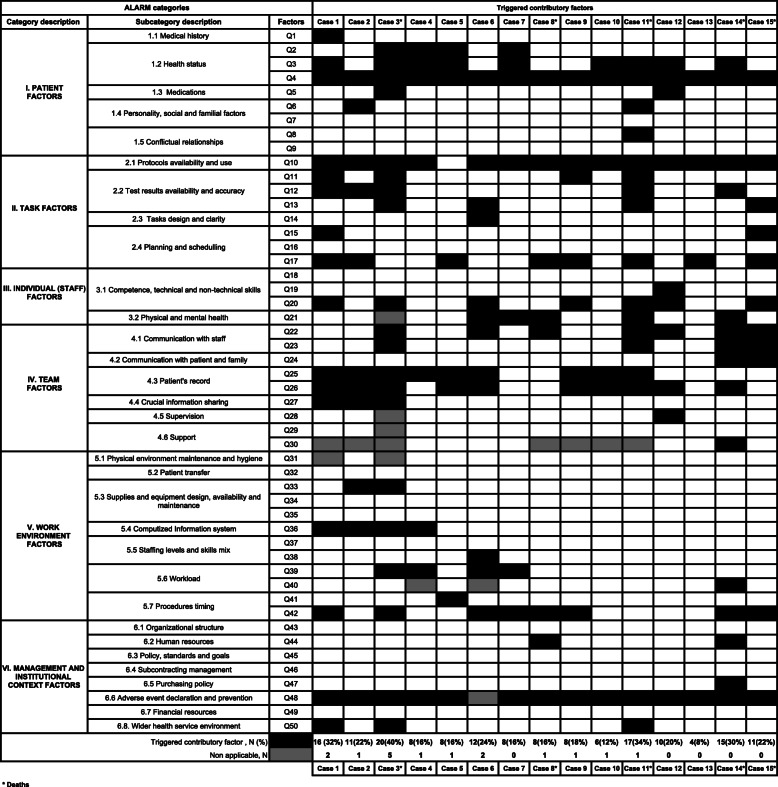
Combinations of triggered contributory factors across the 15 single cases
- Change the below author names:

- Mohammed Anass **Majbar**

- Laila **Amrani**

- Abdelilah **Ghannam**

The original article has been updated as well.
