# Supplementary material for: Contributing factors to severe complications after liver resection: an aggregate root cause analysis in 105 consecutive patients
Source: Patient Saf Surg. 2020 Sep 29;14:36. doi: 10.1186/s13037-020-00261-7 (PMC7526378; doi:10.1186/s13037-020-00261-7)
Supplement: Supplementary file 1 — Additional file 1. Commented ALARM framework as proposed by the French High Authority for Health. [file 13037_2020_261_MOESM1_ESM.pdf]

Grille ALARM Commentée

1. Facteurs liés au patient

Dans toutes les situations cliniques, l'état de santé du patient aura la plus directe influence sur la pratique et les résultats. D'autres facteurs comme la personnalité, le langage et toutes incapacités peuvent aussi être important car ils peuvent influencer la communication avec l'équipe et augmenter la probabilité d'occurrence d'un évènement
Ceci est renforcé auprès des populations présentant une vulnérabilité et des risques particuliers : les personnes âgées ; les patients porteurs de maladies chroniques ; les enfants et adolescents ; les personnes atteintes d'un handicap ; les personnes démunies ; les personnes détenues.

1,1 Antécédents

Exemples	Médicaux<br>habitus
Questions	Les antécédents médicaux du patient ont-ils influencé le cours de l'événement ?

1,2 Etat de santé (pathologies, co-morbidités)

Exemples	Complexité, gravité<br>Personnes vulnérables: personnes âgées, enfants, personnes handicapées…
Questions	Est-ce que l'âge du patient, la gravité de son état ou la complexité de son cas ont pu contribuer à la survenue de cet événement ?<br>Quel était le pronostic vital ou fonctionnel du patient au moment de l'acte ?

1,3 Traitements

Exemples	Risques connus associés aux traitements<br>Modalités particulières d'hospitalisation (détenus, hospitalisation sans consentement...)
Questions	Le patient présentait-il un risque connu ayant influencé l'événement (ex : un traitement particulier) ?

1,4 Personnalité, facteurs sociaux ou familiaux

Exemples	Problèmes de compréhension, d'expression orale, barrière de la langue<br>Qualité de l'entourage, position familiale particulière, métier particulier<br>Personnes démunies, personnes détenues
Questions	Le patient avait-il des problèmes d'expression ? Une communication difficile ?<br>La langue parlée et comprise par le patient était-elle une difficulté lors de la prise en charge ?<br>Des facteurs sociaux ont-ils participé à la survenue de l'événement ?<br>Est-ce que le patient/son entourage était utile et coopératif ?

1,5 Relations conflictuelles

Exemples	Mauvaise qualité de la relation du patient envers les soignants et les soins. Influence négative liée à une hospitalisation antérieure, à l'histoire de la personne ou sa représentation du monde hospitalier.<br>Comportements: agressivité, irrespect, méfiance, opposition, absence d'écoute ou indifférence aux soins
Questions	Quelle était la relation du patient avec les soignants et les soins ?<br>Quelle était l'implication du patient dans la prise en charge thérapeutique (indifférent, opposant, ..) ?

2. Facteurs liés aux tâches à accomplir

La définition ainsi que la planification adéquate sont des facteurs de sécurité des tâches à accomplir. Les protocoles et les procédures permettent de définir les tâches et les processus ainsi que les compétences nécessaires pour leur réalisation.

2,1 Protocoles (indisponibles, non adaptés ou non utilisés)

Exemples	Absence, indisponibilité, inadaptation de protocoles auprès du personnel<br>Mauvaise qualité de l'information dans les protocoles<br>Méconnaissance, oubli de l'existence de protocoles, refus d'utilisation<br>(Il peut s'agir de protocoles diagnostics, thérapeutiques, de soins, organisationnels...)
Questions	Existe-t-il des protocoles en rapport avec les actes ou le processus en cause dans l'événement ?<br>Si les protocoles existent, sont-ils connus, disponibles et utilisés ?<br>Les protocoles sont-ils toujours d'actualité ?

2,2 Résultats d'examens complémentaires (non disponibles ou non pertinents)

Exemples	Difficulté de réalisation ou non réalisation d'un examen (indisponibilité des secteurs médico-techniques...)<br>Difficulté d'accès ou indisponibilité des résultats d'examens<br>Délais de transmission des résultats inadaptés à l'état clinique du patient<br>Doutes sur la fiabilité ou la pertinence des résultats (transmission orale, incomplète ou de mauvaise qualité, défaut d'identification du patient)<br>Désaccords ou doutes sur l'interprétation des résultats d'examens (défaut d'avis spécialisé...)
Questions	Les examens complémentaires ont-ils été réalisés et les résultats disponibles en temps utile ?<br>Existe-il un accord (consensus) concernant l'interprétation des résultats d'examens ?

2,3 Aides à la décision (équipements spécifiques, algorithmes décisionnels, logiciels, recommandations)

Exemples	Absence ou insuffisance d'utilisation de moyens techniques lors de la prise de décision : équipement spécifique, algorithme décisionnel, logiciel, recommandations, reminder…
Questions	Les moyens nécessaires à une prise de décision existent-ils ?, Sont-ils disponibles ?, Ont-ils été utilisés ?

2,4 Définition des tâches

Exemples	Définition des tâches imprécise (quel personnel, quelle compétence, quel acte, quel délai et pour quel résultat) ou absence de définition
Questions	Les tâches concernées étaient-elles bien définies ?<br>La définition des tâches prend-t-elle en compte les compétences des différentes professions ?<br>La définition des tâches est-elle connue, partagée, respectée dans l'équipe ?<br>Existe-t-il une incompréhension de la part du personnel sur les tâches à effectuer ?

2,5 Programmation, planification

Exemples	Défaut de programmation opératoire (absence, modification...)<br>planification des soins ou des tâches non adaptée
Questions	Les tâches concernées étaient-elles planifiées ?<br>L'intervention était-elle programmée ? Le programme a-t-il été respecté, modifié ?<br>La personne appropriée a-t-elle été consultée quand cela était nécessaire pour le déroulement des soins ?

3. Facteurs liés à l'individu (soignant)

Les facteurs individuels comprennent la connaissance, la qualification, et l'expérience de chaque membre de l'équipe et vont affecter leur pratique clinique

3,1 Qualifications, compétences

Exemples	Inadéquation des qualifications et des compétences (savoir-faire) ou des connaissances (savoir)<br>Manque d'entrainement à des situations particulières (urgences vitales, gestes particuliers ...)<br>Manque de motivation
Questions	Pensez-vous que vous aviez suffisamment de connaissances et d'expérience pour prendre en charge ce problème, cette complication ?<br>Aviez-vous déjà réalisé ce geste, cette procédure ?

3,2 Facteurs de stress physique ou psychologique

Exemples	Mauvaise disposition physique et mentale (ex: effets de la charge de travail, de la maladie, etc. sur l'état psychologique et physique des individus)
Questions	Vous sentiez-vous fatigué, affamé ou malade ?<br>Etiez-vous stressé ?

4. Facteurs liés à l'équipe

Chaque professionnel est membre d'une unité, d'une équipe, et plus largement de l'hôpital. La façon dont un professionnel travaille et son impact sur le patient est contraint et influencé par les autres membres de l'équipe et par la façon dont ils communiquent entre eux, s'assistent, s'organisent et se contrôlent
La relation thérapeutique qui s'établit entre une équipe et un patient est un facteur influençant la qualité des soins

4,1 Communication entre professionnels

Exemples	Défaut de communication dans l'équipe ou en dehors du service, du département; entre juniors et seniors au sein de l'équipe; entre différentes professions; entre professionnel du même niveau<br>Difficultés d'expression des désaccords ou des préoccupations<br>Conflit ou mauvaise ambiance au sein de l'équipe<br>Déficience des mécanismes de coordination permettent d'assurer le relais entre les équipes.
Questions	La communication entre vous-même et les autres membres de l'équipe est-elle effective ?<br>La communication est-elle précise, complète et non ambigüe ? Utilise-t-elle un vocabulaire standard (commun) et pas de jargon ?<br>Est-il possible d'exprimer des désaccords ou des préoccupations au sein de l'équipe ? Existe-t-il un temps ou un espace pour cela ?<br>La collaboration et l'ambiance sont-elles satisfaisantes ?<br>Comment qualifieriez-vous la qualité relationnelle dans l'équipe de travail (ambiance, existence de conflits...) ?<br>Est-ce que la communication entre votre service (département ou pôle) et les autres services (départements ou pôles) est effective ?

4,2 Communication vers le patient et son entourage

Exemples	Insuffisance d'échange d'informations entre les professionnels et le patient ou son entourage<br>Défaut de qualité de la relation avec le patient ou son entourage
Questions	L'équipe a t'elle eu (passé) suffisamment de temps avec le patient pour lui expliquer les procédures et les conséquences possibles ou complications ?<br>Ya t-il eu des difficultés linguistiques, culturelles ou des incompréhensions entre l'équipe et le patient et son entourage ?<br>Quelles-sont les habitudes du service en termes d'information du patient et de son entourage ?

4,3 Informations écrites (dossier patient...)

Exemples	Absence, indisponibilité, incomplétude ou qualité insuffisante des informations écrites (dossier patient, comptes-rendus, fiches...)<br>Divergences dans les écrits<br>Illisibilité et manque de signature des documents
Questions	Les dossiers des patients (supports d'informations) sont-ils accessibles, lisibles, identifiés, et complets ?<br>Les dossiers des patients (supports d'informations) mettent-ils suffisamment en évidence les facteurs de risques ?<br>Quel est le niveau de partage des informations écrites dans l'équipe (nombre de supports, confidentialité...) ?

4,4 Transmissions et alertes

Exemples	Défaut de transmissions orales ou écrites<br>Manque d'informations cruciales
Questions	Comment sont rapportées et partagées les informations cruciales pour la prise en charge du patient entre professionnels ?<br>Les informations sur l'évaluation des patients sont-elles partagées et utilisées par les membres de l'équipe de soins en temps utile ?

4,5 Répartition des tâches

Exemples	Incohérence ou déséquilibre de la répartition des tâches dans l'équipe<br>Glissement de tâches<br>Soins ne relevant pas du champ d'expertise du service
Questions	Comment s'organise le travail entre les membres de l'équipe ?<br>L'équipe est-elle d'accord sur la répartition des tâches ? A-t-il été défini qui prend en charge le patient ou réalise un acte particulier ?

4,6 Encadrement, Supervision

Exemples	Défaut d'encadrement<br>Défaut de coordination dans le service<br>Définition non claire des responsabilités dans l'équipe<br>Défaut de supervision ou de réponse à une demande de soutien<br>Supervision inadaptée des médecins et des autres personnels<br>Indisponibilité ou manque de réactivité des seniors (ou référents)<br>Difficulté dans la recherche d'information auprès d'un autre professionnel (avis spécialisés...)
Questions	Avez-vous eu un encadrement (supervision) ou un soutien suffisant ?<br>Un conseil ou l'aide d'un autre membre de l'équipe étaient-ils disponibles tout le temps ?<br>La communication entre le management/supervision et l'équipe de soins est-elle adéquate ?<br>Les coordonnées des spécialistes à appeler en cas d'urgence vitale sont-elles disponibles dans le secteur d'activité ?

4,7 Demandes de soutien ou comportements face aux incidents

Exemples	Manque de soutien par les pairs après un incident<br>Manque de soutien entre différentes professions (ex : cadre soignant et jeune médecin)<br>Faible propension des juniors à solliciter de l'aide<br>Défaut d'adaptation à une situation imprévue
Questions	Quels soutiens sont disponibles en cas de problèmes ?<br>Avez-vous eu un soutien suffisant ?<br>Existe-t-il un soutien effectif dans l'équipe ?

5. Facteurs liés à l'environnement de travail

L'environnement de travail au sein d'une unité ou d'un département, qu'il soit physique (locaux, matériels, fournitures...) ou organisationnel (disponibilité de ressources humaines qualifiées, organisation du travail, horaires...), conditionne la qualité et la sécurité des soins dispensés par une équipe.

5,1 Administration

Exemples	Lourdeur du fonctionnement administratif du secteur de soins<br>Difficultés de révision des processus administratifs
Questions	Est-ce que les règlements et les procédures administratifs sont communiqués de façon adéquate ?

5,2 Locaux (fonctionnalité, maintenance, hygiène...)

Exemples	Défauts des bâtiments et de l'environnement (conception, fonctionnalité, espace, température, lumière, ...)<br>Défaut de maintenance des locaux<br>Hygiène insuffisante des locaux ou défaillance du nettoyage
Questions	Est-ce que votre pratique a été affectée par l'environnement de travail (chaleur, bruit..) ?<br>Est-ce que les locaux sont adaptés au type de prise en charge ?

**5,3**	**Déplacements, transferts de patients entre unités ou sites**
Exemples	Indisponibilité, lenteur ou qualité du brancardage et des transferts<br>Non respect des règles d'hygiène et de sécurité lors des transports<br>Non respect de la dignité, de la confidentialité et du confort du patient
Questions	Les modalités de déplacement du patient ont-elles participé à la survenue de l'événement ?<br>Des circuits et des modes de transport spécifiques ont-ils été définis pour les différents types de prise en charge (hospitalisation complète programmée, chirurgie ambulatoire, urgences immédiates, urgences différées).
**5,4**	**Fournitures ou équipements (non disponibles, inadaptés ou défectueux)**
Exemples	Dysfonctionnements des équipements et approvisionnements<br>Fourniture ou équipement non disponible, inadapté ou défectueux (ex: matériel d'urgence...)<br>Fourniture ou équipement mal utilisés (défaut de formation des personnels, défaut de notices explicatives...)<br>Défaut de maintenance des équipements<br>Absence d'équipement de secours, de solution dégradée ou de dépannage d'urgence permettant de répondre à une panne d'un équipement biomédical critique<br>Fonctionnalité insuffisante des équipements (ex : ergonomie, conception, sécurité, normalisation)<br>Défaut de stérilisation
Questions	Avez-vous disposé des fournitures ou des matériels médicaux nécessaires ?<br>Est-ce que tous les équipements que vous avez utilisé ont fonctionné de façon adéquate et efficacement ?<br>Est-ce qu'il y avait une information suffisante et fiable concernant tous les équipements ?<br>Comment a été assurée la formation des professionnels à l'utilisation de ce matériel ?<br>L'établissement dispose t-il d'un programme de maintenance ?<br>Comment est assuré le dépannage d'urgence des équipements en panne, notamment concernant les dispositifs biomédicaux critiques ? (équipement de secours, solution dégradée ou dépannage d'urgence) Cette procédure est-elle connue des professionnels ?
**5,5**	**Informatique (disponibilité, fonctionnement, maintenance)**
Exemples	Défaut de communication des systèmes d'information entre eux<br>Inaccessibilité d'informations numérisées ou doutes sur la qualité de l'information<br>Mauvaise gestion des accès informatiques<br>Défaut de maintenance (délais excessifs de dépannage…)<br>Bugs informatiques
Questions	Le système d'information est-il adapté aux orientations stratégiques de l'établissement, du service ?<br>Le système d'information facilite t-il l'accès en temps utile à des informations valides ?<br>Le système d'information aide t-il les professionnels dans leur processus de décision ?<br>Existe-t-il des difficultés de fonctionnement du système d'information ?<br>Existe-t-il plusieurs systèmes d'information ?<br>Le système d'information du bloc opératoire est-il intégré au système d'information hospitalier ?
**5,6**	**Effectifs (adaptés en nombre ou en compétences)**
Exemples	Mauvaise combinaison des compétences ou des effectifs<br>Mauvaise adaptation au poste de travail<br>Absence de dispositions pour assurer la sécurité de la prise en charge du patient dans les cas où les effectifs nécessaires ne sont pas présents<br>Indisponibilité d'un personnel apte à exécuter correctement et dans les temps une tâche spécifique<br>Mauvaise gestion des gardes et astreintes
Questions	La composition de l'équipe était-elle appropriée ?<br>Des règles de présence ainsi qu'un système de gardes et astreintes sont-ils définis afin d'assurer la permanence des soins 24h/24 ? Si oui, ces informations sont-elles connues des professionnels ?
**5,7**	**Charge de travail, temps de travail**
Exemples	Irrégularité des coupures ou des temps de repos<br>Charge de travail inadaptée ou trop importante (cumul de gardes, volume des soins, nombre de patients...)<br>Participation à des activités ou des à tâches obligatoires sans lien avec le poste occupé (travail administratif...)
Questions	Avez-vous eu une augmentation de la charge de travail non prévue ou soudaine ?<br>Avez-vous dû faire face à (ou hiérarchiser) plus d'un cas en même temps ?<br>Deviez-vous passer du temps à des activités non cliniques ?
**5,8**	**Retards, délais**
Exemples	Précipitation dans la réalisation d'actes<br>Influence néfaste de délais imposés (inclusion dans un programme opératoire...)<br>Mauvaise appréciation de la notion d'urgence (urgences immédiates, urgences différées...)
Questions	Y a-t-il eu des retards dans la mise en œuvre des procédures de soins ?

6. Facteurs liés à l'organisation et au management

L'équipe est influencée par les actions de management et les décisions prises à un niveau supérieur dans l'organisation. Ceci inclut les politiques de remplacement, d'utilisation de personnel intérimaire, de formation continue, d'intégration et d'évaluation, les politiques de gestion des équipements, mais également la politique sur la qualité et la gestion des risques

6,1 Structure hiérarchique (organigramme, niveaux décisionnels)

Exemples	Structure hiérarchique de l'organisation imprécise (méconnaissance de l'organigramme)<br>Degré d'autonomie du personnel insuffisant<br>Niveaux décisionnels trop importants, inefficaces<br>Changements récents d'organisation interne
Questions	Comment la structure hiérarchique ou des niveaux décisionnels trop nombreux ont-ils influencés négativement le cours de l'événement ?<br>Les circuits de décision et de délégation sont-ils définis, diffusés et connus des professionnels ?

6,2 Gestion des ressources humaines, intérim, remplaçant

Exemples	Gestion du personnel inadéquate<br>Description des postes insuffisante<br>Insuffisance de gestion prévisionnelle des emplois et des compétences.<br>Les besoins en compétences et effectifs des secteurs d'activité ne sont pas identifiés<br>Absence d'intégration des nouveaux arrivants
Questions	Les compétences nécessaires à une fonction ou à un service sont elles identifiées ?<br>Existe-t-il une organisation afin d'intégrer tout nouvel arrivant dans l'établissement (information sur l'établissement et le secteur d'activité, lui permettant l'exercice de sa fonction) ?<br>Pensez-vous que votre période d'adaptation à l'hôpital/spécialité/service vous a préparé à cette situation ?<br>Avez-vous du collaborer avec un nouveau médecin ou personnel de soins (intérim) avec qui vous n'aviez jamais travaillé ?

6,3 Politique de formation continue

Exemples	Formation ou entraînement insuffisant du personnel<br>Politique de formation continue insuffisante (analyse des besoins et offre de formation insuffisantes, pas de plan de formation...)
Questions	Existe-t-il un plan de formation continue établi en accord avec les besoins des services ?<br>Les actes ou procédures de soins nécessitant une formation ou un entrainement sont-ils identifiés ?

6,4 Gestion de la sous-traitance

Exemples	Insuffisance de la prestation sous-traitée
Questions	Comment la fonction sous-traitée est-elle intégrée dans le fonctionnement du service et de l'équipe ?

6,5 Politique d'achat

Exemples	Influence de la politique d'achat sur la disponibilité ou la qualité des produits (livret thérapeutique, listes limitatives de commande…)<br>Rupture de stock ou d'approvisionnement
Questions	Existe-t-il une politique d'achat ou d'approvisionnement prenant en compte les besoins des services, des utilisateurs et des patients ?<br>Comment sont assurés les approvisionnements en situation normale, en urgence, les jours fériés, les week-ends ?

6,6 Management de la qualité, sécurité, hygiène et environnement

Exemples	Gestion documentaire, procédures de révision et de mise à jour des protocoles, rédaction et suivi de l'utilisation des protocoles<br>Hygiène et sécurité au travail insuffisantes<br>Culture de sécurité insuffisante<br>Sécurité et gestion des risques non perçues comme des objectifs importants<br>Absence de stratégie concourant à sensibiliser et à impliquer les professionnels dans les démarches de sécurité des soins
Questions	Comment qualifieriez-vous la culture sécurité de l'établissement ?<br>Existe-t-il une politique d'amélioration de la qualité et de la sécurité des soins diffusée dans l'établissement et connue des professionnels ?<br>L'établissement évalue t-il et hiérarchise t'il les risques dans les secteurs d'activité ?<br>Existe-t-il des plans d'actions d'amélioration de la qualité et de la sécurité des soins mis en oeuvre dans le secteur d'activité ?<br>Existe-t-il une gestion documentaire dans l'établissement ? Si oui, la gestion documentaire définit-elle les modalités de rédaction, de diffusion et de révision des documents ?<br>Les situations mettant en jeu la sécurité des biens et des personnes sont-elles identifiées ?<br>Existe-t-il un document unique établi sur les conditions de travail des personnels ? Si oui, un plan d'amélioration des conditions de travail est-il défini ?

**6,7**	**Ressources financières**
Exemples	Influence néfaste de la politique ou de problèmes financiers sur le fonctionnement de l'établissement
Questions	Existe-t-il des facteurs financiers ayant influencé cet événement ?<br>L'établissement décline t-il en interne des objectifs prévisionnels de dépenses et s'il y a lieu de recettes ?

7. Facteurs liés au contexte institutionnel

L'organisation elle-même est influencée par le contexte institutionnel, la réglementation et le contexte économique et politique du secteur.

**7,1**	**Politique de santé publique nationale**
Exemples	Influence de la politique de santé sur le fonctionnement de l'établissement<br>Contraintes réglementaires
Questions	L'établissement fait-il l'objet actuellement de mesures de la part d'un organe de l'état ?<br>Existe-t-il des contraintes réglementaires en vigueur pour cet établissement ayant influencé le déroulement de l'événement ? (CPOM,…)
**7,2**	**Politique de santé publique régionale**
Exemples	Ressources sanitaires insuffisantes ou défectueuses (CPOM...)<br>Faiblesse des échanges ou des relations avec les autres établissements (réseaux, coopérations...)
Questions	L'établissement a-t-il mis en place des partenariats en cohérence avec les pathologies qu'il prend en charge ?<br>L'établissement est-il organisé en relation avec d'autres établissements pour le type de prise en charge concerné par l'événement ?<br>Dans l'affirmative, le personnel connaît-il les modalités de cette organisation ?
**7,3**	**Systèmes de signalement**
Exemples	Absence de culture de signalement des situations dangereuses (vigilances...)<br>Absence d'apprentissage par le retour d'expérience des événements indésirables
Questions	L'événement s'est-il déjà produit dans l'établissement ?<br>Le personnel déclare-t-il les événements qu'il rencontre ?<br>Comment est organisé l'établissement pour recueillir et analyser les événements indésirables ?
