# Supplementary material for: Contributing factors to severe complications after liver resection: an aggregate root cause analysis in 105 consecutive patients
Source: Patient Saf Surg. 2020 Sep 29;14:36. doi: 10.1186/s13037-020-00261-7 (PMC7526378; doi:10.1186/s13037-020-00261-7)
Supplement: Supplementary file 2 — Additional file 2. The 50 questions of the MMR reporting tool and their categorization according to the ALARM framework. [file 13037_2020_261_MOESM2_ESM.pdf]

ALARM framework questionnaire		Questions	
ALARM Category	Subcategory description	Code	Description
I. PATIENT FACTORS	1.1 Medical history	Q1	Could the patient's surgical history have influenced the course of events?
	1.2 Health status	Q2	Could the patient's age have contributed to the occurrence of the adverse event?
		Q3	Did the patient's general health condition contribute to the occurrence of the adverse event?
		Q4	Could the technical complexity of the case have contributed to the occurrence of the adverse event?
	1.3 Medications	Q5	Was the patient taking any particular medication that could have influenced the course of events?
	1.4 Personality, social and familial factors	Q6	Did the patient have any expression or communication problems that may have influenced the course of events negatively?
		Q7	Did any social or familial factors contribute to the occurrence of the adverse event?
	1.5 Conflictual relationships	Q8	Did the patient's relationship with the healthcare team present a difficulty in the healthcare management?
		Q9	Did the relationship of the patient's entourage with the healthcare team present a difficulty in the healthcare management?
II. TASK FACTORS	2.1 Protocols availability and use	Q10	Are there operational protocols related to the acts or process involved in the event?
	2.2 Test results availability and accuracy	Q11	Have adequate additional medical tests been carried out?
		Q12	Were the results of the requested medical tests available on time?
		Q13	Was there any doubt as to the interpretation of the results of the tests requested?
	2.3 Tasks design and clarity	Q14	Were the tasks well defined and adapted to each member of the team according to his/her professional competence?
	2.4 Planning and schedulling	Q15	Was the treatment strategy planned?
		Q16	Was the surgery scheduled?
		Q17	Was the scheduled surgery carried without intraoperative changing the strategy?
III. INDIVIDUAL (STAFF) FACTORS	3.1 Competence, technical and non-technical skills	Q18	Did the treating doctor/nurse have enough knowledge and experience to perform the medical/surgical procedure?
		Q19	Did the treating doctor/nurse who managed the complication have enough knowledge and experience to manage it?
		Q20	Could a request for additional support (advice or help) have had a positive influence on the course of events?
	3.2 Physical and mental health	Q21	Did the staff members feel tired, stressed, hungry, or sick while handling the case?
IV. TEAM FACTORS	4.1 Communication with staff	Q22	Could a more effective communication between the members of the team have had a positive influence on the course of events (e.g: resident/attending; nurse/doctor, anesthesiologist/surgeon)?
		Q23	Could a more effective communication between the team and the other departments have had a positive influence on the course of events?
	4.2 Communication with patient and family	Q24	Could a more effective communication with the patient and his entourage positively influence the course of events?
	4.3 Patient's record	Q25	Was the patient's record accessible, readable, identified, and complete?
		Q26	Were the risk factors sufficiently highlighted in the patient'record ?
	4.4 Crucial information sharing	Q27	Was crucial information regarding patient management shared between professionals in a timely manner?
	4.5 Supervision	Q28	Was the healthcare team's supervision sufficient?
	4.6 Support	Q29	Was support (advice or help) in dealing with an incident or difficulty available and sufficient?
		Q30	Was moral support after an incident available and sufficient?
V. WORK ENVIRONMENT FACTORS	5.1 Physical environment maintenance and hygiene	Q31	Did the work environment (poor hygiene, heat, noise, etc.) contribute negatively to the occurrence of the adverse event?
	5.2 Patient transfer	Q32	Did the patient's transportation arrangements contribute negatively the occurrence of the adverse event?
	5.3 Supplies and equipment design, availability and maintenance	Q33	Were the supplies or equipment unavailable, unsuitable or defective?
		Q34	Have the supplies or equipment been misused?
		Q35	Did a failure to sterilize the equipment contribute to the occurrence of the adverse event?
	5.4 Computized Information system	Q36	Are there any difficulties with the functioning of the information system that may have contributed to the occurrence of the adverse event?
	5.5 Staffing levels and skills mix	Q37	Was the size (number) of the team appropriate when the event occurred?
		Q38	Was the composition (skills) of the team appropriate when the event occurred?
	5.6 Workload	Q39	Did an increase in the clinical workload (multiple on-call shifts, number of patients...) have an influence on the event?
		Q40	Did the non-clinical workload (administrative tasks...) have an influence on the event?
	5.7 Procedures timing	Q41	Was the execution of the medical/surgical acts precipitated by imposed deadlines?
		Q42	Have there been any delays in complication managment ?
VI. MANAGEMENT AND INSTITUTIONAL CONTEXT FACTORS	6.1 Organizational structure	Q43	Did the presence of too many decision-making levels or complex decision-making levels negatively influence the course of the event?
	6.2 Human resources	Q44	Could a failure to train a newcomer in the establishment have had a negative influence on the event?
	6.3 Policy, standards and goals	Q45	Could a lack of ongoing education have negatively influenced the event?
	6.4 Subcontracting management	Q46	Did an element linked to the outsourced services have a negative impact on the event?
	6.5 Purchasing policy	Q47	Did a shortage or supply problem have a negative impact on the event?
	6.6 Adverse event declaration and prevention	Q48	If the event has already occurred in the establishment, has it been the subject of a declaration and of specific improvement measures?
	6.7 Financial resources	Q49	Are there financial factors that negatively influenced the event?
	6.8. Wider health service environment	Q50	Are there any regulatory constraints in force for the establishment that negatively influenced the progress of the event?
